# Wealth-related versus income-related inequalities in dental care use under universal public coverage: a panel data analysis of the Japanese Study of Aging and Retirement

**DOI:** 10.1186/s12889-015-2646-9

**Published:** 2016-01-12

**Authors:** Keiko Murakami, Hideki Hashimoto

**Affiliations:** 1 Department of Hygiene and Public Health, School of Medicine, Teikyo University, 2-11-1 Kaga, Itabashi-ku, Tokyo 173-8605 Japan; 2 Department of Health and Social Behavior, School of Public Health, The University of Tokyo, 7-3-1 Hongo, Bunkyo-ku, Tokyo 113-0033 Japan

**Keywords:** Dental care, Income, Inequality, Japan, Universal coverage, Wealth

## Abstract

**Background:**

There is a substantial body of evidence of income-related inequalities in dental care use, attributed to the fact that dental care is often not covered by public health insurance. Wealth-related inequalities have also been shown to be greater than income-related inequalities. Japan is one of the exceptions, as the the universal pubic health insurance system has covered dental care. The aim of this study was therefore to compare wealth- and income-related inequalities in dental care use among middle-aged and older adults in Japan to infer the mechanisms of wealth-related inequalities in dental care use.

**Methods:**

Data were derived from the Japanese Study of Aging and Retirement, a survey of community-dwelling middle-aged and older adults living in five municipalities in eastern Japan. Of the participants in the second wave conducted in 2009, we analyzed 2581 residents. Dental care use was measured according to whether the participant had been seen by a dentist or a dental hygienist in the past year. The main explanatory variables were income and wealth (financial assets, real assets and total wealth). The need for dental care was measured using age, the use of dentures and chewing ability. The concentration indices for the distribution of actual and need-standardized dental care use were calculated.

**Results:**

Among the respondents, 47.9 % had received dental care in the past year. The concentration index of actual dental care use (CI) showed a pro-rich inequality for both income and wealth. The CIs for all three wealth measures were larger than that for income. A broadly comparable pattern was seen after need-standardization (income: 0.020, financial assets: 0.035, real assets: 0.047, total wealth: 0.050).

**Conclusions:**

The results showed that wealth-related inequalities in dental care use were greater than income-related inequalities in Japan, where most dental care is covered by the public health insurance system. This suggests that wealth-related inequalities in dental care use cannot be explained by economic budget constraints alone. Further studies should investigate the mechanisms of wealth-related inequalities in dental care use.

## Background

Oral diseases remain a major public health issue for high-income countries, where expenditure on treatment often exceeds that for other diseases [1]. Dental caries and periodontal disease have historically been considered to constitute the most important global oral health burden and the major reasons for tooth extraction [2]. The experience of pain, problems with eating, chewing, smiling, and communication because of missing, discolored, or damaged teeth have a major impact on people’s daily lives and wellbeing [2]. Periodontal disease may also have long-term consequences for general health by increasing the risk of chronic diseases such as diabetes mellitus and cardiovascular diseases [3].

Although dental diseases are largely preventable by routine dental care [4, 5], several factors have been suggested as determinants of dental care use; race, age, sex, marital status, educational level, income, amount and type of health insurance, level of perceived disease burden, and cultural values associated with oral health [6–8]. Among these factors, many studies have consistently shown income-related inequalities in dental care use [9–14], which could at least partially explain socioeconomic inequalities in oral health.

Income-related inequalities in dental care use are often attributed to the limited affordability of dental care, as it is often not covered by public health insurance in many developed countries [7]. Most recently, wealth was reported to be more sensitive than income in predicting socioeconomic inequality in health-related outcomes, especially in older people who are likely to be retired and financially dependent on assets rather than cash income [15, 16]. Greater inequalities caused by wealth rather than income have been found in dental care use in the United States and Europe [17]. Whether the extension of public insurance coverage to dental care would be an effective means of closing these equality gaps has important policy implications for improving oral health regardless of people’s ability to pay.

In this regard, the situation in Japan could provide evidence to inform the debate, as the universal pubic health insurance system has covered dental care as well as medical care since 1961 [18]. As of 2010, the copayment rate has been set at 30 %, reduced to 10 % for people aged 70 years and older. Although some forms of preventive dental care are not covered by public insurance, given the generous benefits available, we hypothesized that economic budget constraints would not play as large a role in Japan as in other developed countries without public dental care coverage. If greater inequalities caused by wealth rather than income exist also in Japan, this could not be explained by economic budget constraints alone. Thus, a financial policy to equalize access to dental care may fall short in reducing inequality. It is said that the roles of wealth go far beyond the ability to pay [19, 20]. Wealth is a more stable indicator of class status in society than income, because wealth can enable a family to achieve and maintain a certain status [21]. Class-related cultural resources serve to manifest social class inequalities of people’s health chances and choices [22, 23]. Therefore, wealth-related inequalities in dental care use may indicate a gap in the class-related norm of oral hygiene, which would require active preventive intervention targeted to vulnerable people. We therefore compared wealth- and income-related inequalities in dental care use among middle-aged and older adults in Japan to infer the mechanisms of wealth-related inequalities in dental care use.

## Methods

### Data and participants

We used data from the Japanese Study of Aging and Retirement (JSTAR), which was conducted by the Research Institute of Economy, Trade and Industry, Hitotsubashi University, and The University of Tokyo. The details regarding JSTAR can be found elsewhere [24]. The JSTAR is a sister survey in a larger international collaboration with the Health and Retirement Study (HRS) in the United States [25] and the Survey of Ageing and Retirement in Europe (SHARE) in Europe [26]. The first JSTAR survey was conducted in five municipalities in eastern Japan between January and April in 2007. The survey sampled community residents aged between 50 and 75 years using probabilistic sampling in each municipality, and 4163 residents filled in the leave-behind questionnaire and were interviewed later (response rate 59.1 %). Of the 3906 participants in the first wave, 3083 participated in the second wave in 2009 (follow-up rate 78.9 %) (see http://www.rieti.go.jp/en/projects/jstar/). We received approval from the Research Institute of Economy, Trade and Industry for the secondary use of 2712 resample of the JSTAR data (both first and second waves), with data anonymized (88 % of second-wave participants). We analyzed 2581 residents with complete values for all the variables used in the analysis other than income and wealth. We used the data measured in the second wave other than the use of dentures and chewing ability.

### Dental care use

Our targeted variable was dental care use in the past year, measured through the self-report question, “In the past year, have you been seen by a dentist or a dental hygienist? Include visits for regular adjustment of dentures or treatment for periodontal diseases.”

### Income and wealth

Income was measured as the total amount of labor income, pension income, capital gains from financial assets, real estate investment, and private transfers during the past year. Equivalent income was defined as annual net household income (the sum of the respondent’s and the spouse’s income if household expenses were managed jointly; otherwise, that of the respondent) divided by the square root of the number of family members [24]. The number of family members included the respondent, his/her spouse, and their economically dependent children [24]. If household expenses were managed separately, we subtracted 1 from the number of family members, since the spouse was considered as heading a different household [24].

We evaluated three types of wealth: financial assets, real assets, and total wealth. Financial and real assets were treated separately because these two assets differ in terms of liquidity (ease of conversion into cash). Financial assets were defined as the sum of deposits, bonds, and stocks minus non-mortgage liabilities. Equivalent net financial assets were computed by dividing the total amount by two if household financial assets were managed jointly by a husband and wife; the total amount was used if the respondent was a single or managed household financial assets separately [24]. Real assets were defined as the value of housing and land minus the current amount of mortgage loans. Equivalent net real assets were computed by dividing the amount by two if and only if a respondent was married on the basis of the assumption that real assets were managed jointly by married couples [24].

Income and wealth had a significant number of missing observations; the percentages of missing observations were 26.7 % for income, 44.1 % for financial assets, 19.1 % for real assets, and 51.5 % for total wealth. Therefore, we applied multiple imputations of income and wealth with multivariate normal regression on other explanatory variables [27]. For a robustness check, we conducted the same analyses before imputation and obtained similar results. Thus, we present only the results with multiple imputations of income and wealth.

### Need and non-need variables related to dental care use

According to the categorizations used by previous studies [9, 11], needs were classed as health-related conditions leading to healthcare use, and non-needs were referred to socioeconomic conditions and preferences that determine healthcare use despite health conditions.

Following a previous study [17], the need for dental care was measured using age, the use of dentures, and chewing ability. Age was categorized into 52–59, 60–64, 65–69, and 70 years and older. The use of dentures and chewing ability measured in the first wave were used to predict the need for dental care in the second wave, as the outcome variable in this study was dental care use in the past year. The use of dentures was measured using the question, “Do you normally wear dentures, including partial dentures or implants?” Chewing ability was determined from a five-response level question: “What level of hard objects can you bite, with dentures or implants if you use them?: I can chew and eat anything I want to; Some things are difficult to chew, but I can eat almost anything; I can’t chew very well, so the foods I can eat are limited; I can hardly chew at all; I can’t chew at all, and eat blended foods only.” Following a previous study in Japan [28], the last three categories were combined because of the small number of respondents. As a result, chewing ability was categorized as “very well”, “fairly well”, and “not well”.

Referring to previous studies, we included sex, marital status, educational attainment, and work status as non-need variables related to dental care use [11, 12, 17, 29]. Insurance status was not included because the same benefit is applied to all adult residents aged under 70 in Japan (except for those under welfare programs) and private dental insurance did not exist at that time.

### Data analysis

Spearman’s correlation coefficients were calculated between income and wealth. Differences in the proportions of dental care use by need and non-need variables were assessed by chi-square test. The concentration index method was used to quantify the degree of socioeconomic inequality in dental care use [30–32]. The basic idea of a concentration index resembles a Lorenz curve method, where the accumulated care use is plotted along the rank of socioeconomic resources on the horizontal axis, and the concentration index is twice as large as the area between the curve and the diagonal line. Positive values indicated a pro-rich inequality (disproportionate concentration of dental care use among the rich), whereas negative values indicated the opposite. The concentration indices for the distribution of actual use and need-standardized use were calculated, which were called the CI (concentration index) and the HI (horizontal inequity index), respectively. Using a probit model, we obtained the estimated likelihood of dental care use after standardizing the need variables set in mean values [32]. The HI should reflect those inequalities solely attributed to non-need variables such as socioeconomic conditions [11, 12, 17, 29]. In addition to the concentration index method, we conducted multiple logistic analyses, adjusting for need and non-need variables to evaluate the importance of separate independent variables in explaining dental care use.

These procedures were conducted for income, financial assets, real assets, and total wealth. All analyses were performed using Stata version 12 (StataCorp LP, College Station, TX, USA). *P*-values less than 0.05 were considered statistically significant.

## Results

The respondent’s characteristics are presented in Table 1. Among the respondents, 51.0 % were men, 81.0 % were married under formal or common law, 22.6 % were educated beyond senior high school, 52.3 % were currently in paid work, 50.0 % wore dentures, and 96.1 % reported being able to chew foods “very well” or “fairly well.” Table 2 shows the Spearman’s correlation coefficients among income and wealth. The correlation coefficients ranged from about 0.20 to 0.30, except for the correlation between total wealth and financial/real assets.

**Table 1:** Characteristics of respondents: the Japanese Study of Aging and Retirement (JSTAR), 2009

			Number	Percent
Need variables				
	Age			
		52–59 years	588	(22.8)
		60–64 years	537	(20.8)
		65–69 years	585	(22.7)
		≥70 years	871	(33.7)
	Use of dentures^a^			
		Yes	1290	(50.0)
		No	1291	(50.0)
	Chewing ability^a^			
		Very well	1556	(60.3)
		Fairly well	924	(35.8)
		Not well	101	(3.9)
Non-need variables				
	Sex			
		Men	1317	(51.0)
		Women	1264	(49.0)
	Marital status			
		Married/Common-law	2090	(81.0)
		Others	491	(19.0)
	Educational attainment			
		Elementary/Junior high school	908	(35.2)
		Senior high school	1090	(42.2)
		>Senior high school	583	(22.6)
	Work status			
		Working	1349	(52.3)
		Not working	1232	(47.7)

^a^Data on use of dentures and chewing ability were derived from the first wave of JSTAR, 2007

**Table 2:** Spearman’s correlation coefficients among income and wealth, 2009

	Income	Financial assets	Real assets	Total wealth
Income				
Financial assets	0.260			
Real assets	0.220	0.205		
Total wealth	0.291	0.595	0.884	

All correlation coefficients were statistically significant (*p*-value < 0.001)

Table 3 shows the proportions of respondents who had undergone dental care according to need and non-need variables. Among the respondents, 1235 (47.9 %) had received dental care in the past year. The probability of dental care use was higher in women than in men, and increased with higher education. Age group, the use of dentures, chewing ability, marital status, and work status were not associated with the probability of dental care use.

**Table 3:** Proportions of dental care use by need and non-need variables, 2009

			Percent	*p*-value^a^
Total			47.9	
Need variables				
	Age			0.237
		52–59 years	45.1	
		60–64 years	48.4	
		65–69 years	50.9	
		≥70 years	47.3	
	Use of dentures^b^			0.678
		Yes	47.4	
		No	48.3	
	Chewing ability^b^			0.608
		Very well	48.6	
		Almost well	46.5	
		Not well	48.5	
Non-need variables				
	Sex			0.047
		Men	45.9	
		Women	49.8	
	Marital status			0.272
		Married/Common-law	48.4	
		Others	45.6	
	Educational attainment			0.001
		Elementary/Junior high school	43.1	
		Senior high school	49.5	
		>Senior high school	52.1	
	Work status			0.408
		Working	47.1	
		Not working	48.7	

^a^ *p*-value was calculated by χ^2^ test

^b^Data on use of dentures and chewing ability were derived from the first wave of JSTAR, 2007

The concentration index (CI) and horizontal inequity index (HI) values for dental care use by income and wealth are shown in Table 4. The CI of actual dental care use showed a pro-rich inequality for income and wealth, although the CI for income was not significantly different from zero. The CI for real assets was larger than the CI for financial assets. A broadly comparable pattern of pro-rich inequality was seen in the HI after need standardization (income: 0.020, financial assets: 0.035, real assets: 0.047, total wealth: 0.050).

**Table 4:** Concentration Index (CI) and Horizontal Inequity Index (HI)^a^ for dental care use

	CI	*p*-value	HI	*p*-value
Income	0.010	0.501	0.020	0.178
Financial assets	0.034	0.019	0.035	0.018
Real assets	0.043	0.004	0.047	0.003
Total wealth	0.046	0.001	0.050	0.001

^a^Concentration index of need-standardized dental care use (standardized using age, use of dentures, and chewing ability)

Table 5 shows multivariate-adjusted odds ratios (95 % confidence intervals) of dental care use in the past year across income/wealth categories. While no significant gradient in dental care use was found across income categories, real assets and total wealth were significantly associated with dental care use. Among need and non-need variables, sex and educational attainment were significantly associated with dental care use.

**Table 5:** Multivariate-adjusted^a^ odds ratio (OR) and 95 % confidence interval (CI) for dental care use

			Income	Financial assets	Real assets	Total wealth
			OR (95 % CI)	OR (95 % CI)	OR (95 % CI)	OR (95 % CI)
Economic variables						
		1st quartile (lowest)	1.00	1.00	1.00	1.00
		2nd quartile	1.03 (0.80–1.31)	1.17 (0.91–1.50)	1.27 (1.00–1.61)	1.28 (0.98–1.67)
		3rd quartile	1.08 (0.82–1.41)	1.25 (0.94–1.66)	1.40 (1.08–1.82)	1.40 (1.07–1.82)
		4th quartile (highest)	0.98 (0.74–1.30)	1.26 (0.96–1.66)	1.40 (1.04–1.86)	1.39 (1.04–1.84)
Need variables						
	Age					
		52–59 years	1.00	1.00	1.00	1.00
		60–64 years	1.22 (0.96–1.54)	1.20 (0.94–1.52)	1.19 (0.94–1.52)	1.18 (0.93–1.51)
		65–69 years	1.42 (1.11–1.82)	1.39 (1.08–1.79)	1.41 (1.10–1.80)	1.37 (1.07–1.76)
		≥70 years	1.29 (1.00–1.66)	1.27 (0.99–1.63)	1.26 (0.98–1.62)	1.24 (0.96–1.60)
	Use of dentures^b^					
		No	1.00	1.00	1.00	1.00
		Yes	1.00 (0.85–1.19)	1.00 (0.84–1.18)	1.01 (0.85–1.19)	1.00 (0.85–1.19)
	Chewing ability^b^					
		Very well	1.00	1.00	1.00	1.00
		Almost well	0.94 (0.79–1.11)	0.94 (0.79–1.12)	0.95 (0.80–1.13)	0.95 (0.80–1.13)
		Not well	1.10 (0.73–1.67)	1.14 (0.75–1.73)	1.14 (0.75–1.73)	1.16 (0.76–1.76)
Non-need variables						
	Sex					
		Men	1.00	1.00	1.00	1.00
		Women	1.20 (1.02–1.41)	1.20 (1.02–1.41)	1.20 (1.02–1.41)	1.19 (1.01–1.40)
	Marital status					
		Married/Common-law	1.00	1.00	1.00	1.00
		Others	0.89 (0.72–1.09)	0.89 (0.73–1.09)	0.95 (0.77–1.18)	0.94 (0.77–1.17)
	Educational attainment					
		Elementary/Junior high school	1.00	1.00	1.00	1.00
		Senior high school	1.36 (1.13–1.64)	1.34 (1.11–1.61)	1.35 (1.12–1.63)	1.33 (1.11–1.61)
		>Senior high school	1.60 (1.27–2.02)	1.55 (1.23–1.95)	1.54 (1.22–1.95)	1.52 (1.21–1.92)
	Work status					
		Working	1.00	1.00	1.00	1.00
		Not working	1.02 (0.85–1.22)	1.02 (0.86–1.22)	1.01 (0.85–1.21)	1.02 (0.85–1.22)

^a^Adjusted for all other variables in the table

^b^Data on use of dentures and chewing ability were derived from the first wave of JSTAR, 2007

## Discussion

The present study found that wealth-related inequalities in dental care use were greater than income-related inequalities in Japan, chiming with the situation reported from countries where dental insurance is seldom provided publicly. Furthermore, the extent of inequality in dental care use by real assets was larger than that by financial assets.

Two previous studies used data sets derived from HRS and SHARE to examine the extent of socioeconomic inequalities in dental care use according to both wealth and income. Among adults aged ≥50 years in the United States and Europe, wealth-related inequalities determined using the concentration index method were consistently greater than corresponding income-related inequalities [17]. The authors thus argued that wealth is more sensitive to household economic conditions than income among those, a large proportion of whom are retired. Using logistic regression analyses of the HRS data set showed that both wealth and income had a strong and positive independent association with dental care use among adults aged ≥50 years in the United States [33]. Because health insurance in the United States scarcely covers dental care expenditure, the authors attributed the inequality to differences in the economic resources of households for out-of-pocket payment [33]. Using corresponding data of community-based middle-aged and older adults in Japan, the present study also found substantial wealth-related inequalities, whereas income-related inequalities were smaller. Despite the difference in the insurance policy in Japan, which covers dental care expenditure with limited copayment, our findings are in keeping with the results of the above studies in the United States and Europe.

There are several possible reasons why wealth-related inequalities in dental care use exist in Japan. First, universal health insurance covers only a limited amount of preventive dental care compared with more comprehensive coverage of curative care. We have previously identified income-related inequality in preventive dental care use among men, while there were no significant income-related inequalities in curative dental care use among working-age Japanese adults [34]. This suggested that inequalities in preventive dental care would lead to wealth-related inequalities in dental care use, as demonstrated in the present study to some degree, while universal coverage seems to function effectively for curative dental care. However, Japanese traditionally take a treatment-oriented approach to dental care [34], while other developed countries place an emphasis on preventive dental care. Furthermore, we previously reported that income-related inequality in preventive dental care was no longer significant after adjusting for covariates among women [34]. Taken together, wealth-related inequalities in dental care use in the present study could not be explained by the relatively higher cost burden of preventive dental care alone.

The other possibility is that the observed wealth-related inequalities in dental care use may not be economic but a non-economic preference for dental care in Japanese context. In a previous study in Europe, the pro-rich inequality in dental care use measured by income was significantly explained by educational attainment, and the authors argued the importance of non-economic preference related to education that determined dental care use [9]. In the present study, wealth may signify factors other than current economic constraint of the middle-aged and older adults, since wealth is the accumulated results of individuals’ socioeconomic status over their life course [19–21], which may relate to a particular life-style that people adopt as seemingly voluntary preferences prevalent in their belonging social strata [22, 23]. People with greater wealth may be more likely to adopt a life-style common among the richer society that are further instrumental in the use of economic resources for health gains [22, 23]. Cultural values associated with oral health have been found to be a determinant of dental care use [8]. Indeed, the rarely life-threatening nature and predictability of demand for dental care may make individual’s care decisions more discretionary [4]. This line of argument is further supported by the result that the extent of inequality in dental care use by real assets was larger than that by financial assets, although real assets had less liquidity (ease of conversion into cash) than financial assets. Socioeconomic inequalities in dental care use are generally discussed from the cost perspective. However, many previous studies showed that social factors other than cost were associated with dental care use [8]. Also, unhealthy lifestyle patterns are in large parts more determined by people’s norms and values than by insufficient financial means [23]. Class-related cultural resources derived from wealth (e.g., health values, perceptions, health knowledge and behavioral norms) would incur wealth-related inequalities in dental care use.

The present findings help us infer some implications for dental care policy. If wealth-related inequality in dental care use is caused by differences in economic resources to purchase dental care, then lowering the economic burden of dental care would narrow this inequality. If non-economic preferences for dental care (e.g., taste and preference for oral health) better explains wealth-related inequality in dental care use, financial support may not fully solve the inequality. Because the present study suggested that these inequalities cannot be fully explained by factors related to the ability to pay, other solutions are required to motivate people to use dental care, regardless of their preference level for dental care and oral health. However, the universal provision of health education programs aimed at enhancing dental care literacy may not reduce but widen the inequality because highly educated people are more responsive to interventions than less educated individuals. Closing the gap demands targeting the vulnerable population. Alternatively, structural and environmental interventions are likely to have an even greater effect on the population [35]. One promising strategy may be to include oral health as a part of general health checks in medical practice, which effect has been demonstrated in the United Kingdom [36]. The Japanese healthcare system has the unique characteristic of including a mandatory annual health check-up for all, but a dental check-up is not included in most settings. In addition, reaching and engaging individuals where they live, work, play, and receive services is an important strategy to ensure high levels of participation in community-based interventions [37].

Some limitations of the present study should be considered. First, income and wealth were based on self-reports, which could be subject to biases due to underreporting or overreporting. However, the measurement of income and wealth in JSTAR was carefully designed following methodological recommendations for accurate self-report measurement [24]. For example, each respondent was asked to fill in the figures in the leave-behind questionnaire by looking at the official tax record kept at home. If respondents did not fill in the income items in the interview, the interviewer asked the respondents to answer these. These methods were also applied when asking about wealth, and the number of categories of wealth (both financial assets and real assets) were large in JSTAR. Also, income and wealth had a significant number of missing observations. In the JSTAR data, those who failed to report their income or wealth were more likely to be married and have a lower educational attainment than those who reported their income and wealth (data not shown). In general, those of lower socioeconomic status are less likely to report their socioeconomic status such as income. Taken together, this might have led to underestimation of the true association. However, as mentioned above, our analysis using multiple imputations of income or wealth showed similar results to the analysis without imputation, making it less likely that non-response on income and wealth substantially affected the results. Second, the type and quality of dental care use (e.g., curative or preventive) could not be examined because of data limitations. Two main measures are often used: the probability of at least one dental care use, and the frequency of dental care use. In previous studies [38, 39], these two types were treated differently because the former is believed to be solely determined by the patient, while the latter may also be affected by other factors such as the provider’s induced demand. Some previous studies have also shown that income is a particularly important determinant for the decision to receive dental care, although this did not drive the frequency of care delivered to those who did receive care [38–40]. Because previous studies using the HRS and SHARE datasets examined wealth-related inequalities in the probability of dental care use [17, 33], we chose the probability of dental care use as an outcome for comparative purposes. Third, although we used panel data because of our concerns with time causality, this might lead to bias due to selective attrition if respondents in the first wave and not included in the second wave were unique in their association between income/wealth and dental care use. For a robustness check, we conducted a similar analysis using cross-sectional data from the second wave; the results (data not shown) did not differ considerably with the results presented here. Finally, the present study did not directly measure other social factors that may determine dental care use. Further studies should examine the effects of possible variables, such as cultural values associated with oral health and taste and preference for preventive care [8].

## Conclusions

The present study showed that wealth-related inequalities in dental care use were greater than income-related inequalities in Japan, where most dental care is covered by the public health insurance system. This is similar to other countries where large proportions—or even the entirety—of adult dental care are excluded from public care insurance. This suggests that wealth-related inequalities in dental care use cannot be explained by economic budget constraints alone. Further studies are needed to investigate the mechanisms of wealth-related inequalities in dental care use.
